# Temporal Trends and Characteristics of Community-Based HIV Testing: First Community-Based Testing Program Results of Men Who Had Sex with Men and Female Sex Workers in Haiti, 2015–2018

**DOI:** 10.4269/ajtmh.24-0391

**Published:** 2025-07-08

**Authors:** Tristan Alain, Maxime Inghels, Jean-Mary Mérisier, Vialli Dimanche, Guillemette Quatremère, Candice Audemard, Sabine Lustin, Charlot Jeudy, Arnoux Descardes, Daniela Rojas Castro, David Michels

**Affiliations:** ^1^ AIDES, Pantin, France;; ^2^ Coalition PLUS, Pantin, France;; ^3^ Lincoln International Institute for Rural Health, University of Lincoln, Lincoln, United Kingdom;; ^4^ Centre Population et Développement (UMR 196 Paris Descartes–IRD), SageSud (ERL INSERM 1244), Institut de Recherche pour le Développement, Paris, France;; ^5^ Volontariat pour le Développement d’Haïti, Port-au-Prince, Haiti;; ^6^ Promoteur objectif zérosida, Port-au-Prince, Haiti;; ^7^ Kouraj, Port-au-Prince, Haiti;; ^8^ SESSTIM, INSERM/IRD/Aix Marseille Université, Marseille, France

## Abstract

To increase HIV status awareness among men who have sex with men (MSM) and female sex workers (FSWs), a community-based HIV testing program was implemented for the first time in Haiti. We aimed to assess 1) the effectiveness of the program to reach HIV-exposed populations over time and 2) the characteristics, HIV exposure, and testing profile of program participants. Rapid diagnostic HIV testing (RDT) was offered to all individuals present in several community-based settings across Haiti. Trends in the number of tested individuals, first-time tested, and HIV positive tested per intervention were described using linear regression. Characteristics and factors related to the first-time test were described. Between July 2015 and April 2018, 445 interventions in 91 community-based venues resulted in 3,998 rapid tests performed. A median of eight individuals was tested per intervention, with 18% first-time testers and 6% with HIV-positive results. The overall numbers of tests and positive test results increased over the intervention program period. Within 1,572 first-time RDT testers (1,216 MSM, 235 FSWs, and 121 nonkey population), 31% (*n* = 489) were first-time HIV testers. First-time tested individuals reported HIV exposure, such as lack of condom use at last intercourse (33%), transactional sex (35%), and recent (≤12 months) sexually transmitted infection (12%). The community-based HIV testing program effectively reached HIV-positive and previously untested individuals over a 3-year period. Continued community-based testing in conjunction with other HIV testing services is recommended for Haiti.

## INTRODUCTION

Although the HIV incidence in Haïti halved between 2010 and 2020 (from 0.91 [0.76–1.10] to 0.45 [0.30–0.62] per 1,000 population), HIV prevalence in the general population remains high (1.9%, 2021). In particular, “key populations,” such as men who have sex with men (MSM) and female sex workers (FSWs), are more affected by HIV (HIV prevalence: 12.9% and 8.7%, respectively, in 2021).^1^ Yet, the majority of key populations living with HIV are not aware of their status. A recent study has shown that only 34% of FSWs and 26% of MSM living with HIV are aware of their status.^2^ Along with common HIV testing access barriers found in the general population,^3^^,^^4^ MSM and FSWs experience additional barriers that prevent them from being HIV tested, such as stigma and gender-based violence,^5^ criminalization of their sexual practices,^6^ and poverty.^7^

To overcome the HIV testing barriers faced by MSM or FSWs, community-based HIV services have been advocated for by the WHO.^8^ Among these services, community-based HIV testing by community-peer educators outside of usual clinical facilities (e.g., mobile clinics and community-based venues) is recommended. Peer educators are trained in the use of rapid diagnostic HIV testing (RDT), which provide HIV test results in <30 minutes.^9^ This type of community-based HIV testing program was also implemented at home and has been shown to be as effective as a mobile approach.^10^ With respect to implementation sites, one study shows that although both community centers and outreach sites are effective, community clinics appear to be more effective.^11^ Community-based HIV services are designed to be nonstigmatizing and client friendly, and they are supported by community-based organizations or nongovernmental organizations (NGOs). Community-based HIV services have been shown to be acceptable and able to reach highly HIV-exposed populations.^9^^,^^12^^–^^14^

In Haiti, the first community-based HIV testing program targeting MSM and FSWs was launched in 2015. This project was coordinated by the Haitian association Volontariat pour le développement d’Haïti in partnership with local communities (e.g., MSM, FSWs, NGOs, and medical staff) with the support of the French association AIDES—the latter having a long track record in developing community-based HIV testing projects.^9^^,^^15^ The program was developed to enhance community-based program relevancy for MSM and FSWs in Haïti and to identify obstacles and levers to reach these exposed populations. This article aims to present the results from this community-based RDT program. More specifically, we aim to assess 1) the effectiveness of the program to reach HIV-exposed populations over the program period and 2) the characteristics, HIV exposure, and testing profile of the individuals participating in the program.

## MATERIALS AND METHODS

### Program design.

A community-based HIV testing program using RDT (third generation rapid HIV ½ INSTI^©^ test; Laboratoires Nephrotek, Boulogne-Billancourt, France) was funded by the French public agency Expertise France from July 31, 2015 to April 11, 2018. The program was implemented in 7 of 10 departments of the Republic of Haiti by four MSM or FSW community-based associations (Volontariat pour le développement d’Haïti, Promoteur Objectif Zérosida, Kouraj, and Plateforme haïtienne pour l’égalité de traitement des personnes). These nonprofit organizations represent the country’s main HIV community-based organizations. The program was designed using a community approach,^16^ which involves communities throughout different stages of the project: for example, conception of the program activities, creation, and validation of a questionnaire (translated in French and Creole) as well as selection of the intervention places.

Areas of intervention were identified and selected by the four community-based associations included in the program. These areas were known for being frequented by MSM and FSW communities. These places included inside locations, such as community-based NGO facilities, and outside locations, such as bars, cartels (i.e., religious voodoo venues where MSM gather), streets, and others places (e.g., stores and religious places), where peer workers offered anonymous free RDT and counseling. Interventions were implemented in areas identified by community associations and frequented by MSM and FSWs. Although the program targeted MSM and FSWs, all individuals frequenting these places were eligible to participate in the intervention.

A pretest questionnaire was administered anonymously face to face to each program participant who accepted to be tested for HIV. Those who were younger than 18 years of age, individuals with psychiatric pathologies, and those who were not able to express informed consent were not offered testing.

This program was the first one to introduce community-based RDT in Haiti.

### Study population.

Two samples were considered in line with the two aims of our study. First, to assess the long-term effectiveness of the program, we considered all completed questionnaires during the program period. Second, because the questionnaires were anonymous and thus, linking two questionnaires to the same individual was not possible, we considered questionnaires where individuals reported having RDT for the first time for the description of the program users. To the best of our knowledge, the absence of other programs offering RDT before or during the study period has limited classification bias.

Because of their low number and for convenience, non-MSM and non-FSW populations (i.e., named the “nonkey population” in the article) that were tested for HIV as part of the program are considered one group.

### Data collected.

A questionnaire was administered anonymously face to face to each participant tested for HIV through the program. The questionnaire was administered in French or Creole by bilingual interviewers. The collection sites coincided with the testing sites. The date and place of collection were recorded.

Each participant was asked 1) whether they have ever been tested for HIV before and 2) whether they have ever been tested by RDT.

We considered the following sociodemographic characteristics: gender, age, city of residence (the capital Port-au-Prince region or other regions), perception of personal financial situation, and sexual partners (men only or men and women). In addition, the number of sexual partners (≤6 months), transactional sex (≤12 months), condom use at last intercourse (yes or no), reported sexually transmitted infection (STI; ≤12 months), testing sites type, reason for undertaking HIV testing, and drug use (≤12 months; excluding alcohol, tobacco, and cannabis) were considered. Perceived risk of acquiring HIV and the result of the RDT were also collected. Male participants reporting male sexual partners were classified as MSM, and female participants reporting transactional sex were classified as FSWs.

## STATISTICAL ANALYSES

To investigate the effectiveness of the program in reaching HIV-exposed populations over time, we compared medians of tested, first-time tested, and HIV-positive tested individuals numbers per intervention every trimester during the program. The percentages of first-time tester and HIV-positive tested individuals were also computed per trimester. Linear regressions were conducted to measure changes in the trends of the number of tests performed over time.

The characteristics, HIV exposure, and testing profiles of the individuals reached by the program were described by population (MSM, FSWs, and nonkey population).

Univariate analysis of associated factors with having a first HIV test within each population was performed. First-time HIV testers and ever tested were compared using a χ^2^ test with the Rao and Scott second-order correction.^17^ Explanatory variables considered included variables related to sociodemographic characteristics, HIV risk exposure and perception, and testing information. Multivariate models were then conducted and included significant variables at the 0.20 threshold from the univariate analysis. A 0.20 threshold was chosen to identify confounders or interaction factors in univariate analyses, and it was included in multivariable models.^18^ A backward procedure based on the Akaike information criteria was used to select significant variables for the final model (*P* <0.05). Individuals with missing data on variables of interest were excluded except on two variables. Because of the number of missing values for condom use at last intercourse (not applicable = 93) and reported STI (not applicable = 439), missing values were included with the “no” answer to avoid data attrition. Selected individual samples were compared with individual samples before exclusion of individuals with missing data using the χ^2^ test.

Analyses were conducted using R v. 4.1.0 (R Foundation, Vienna, Austria),^19^ and cluster effect on intervention sites was implemented using the R package survey.^20^

## RESULTS

### Testing uptake trends during the community-based program.

**Figure 1.**
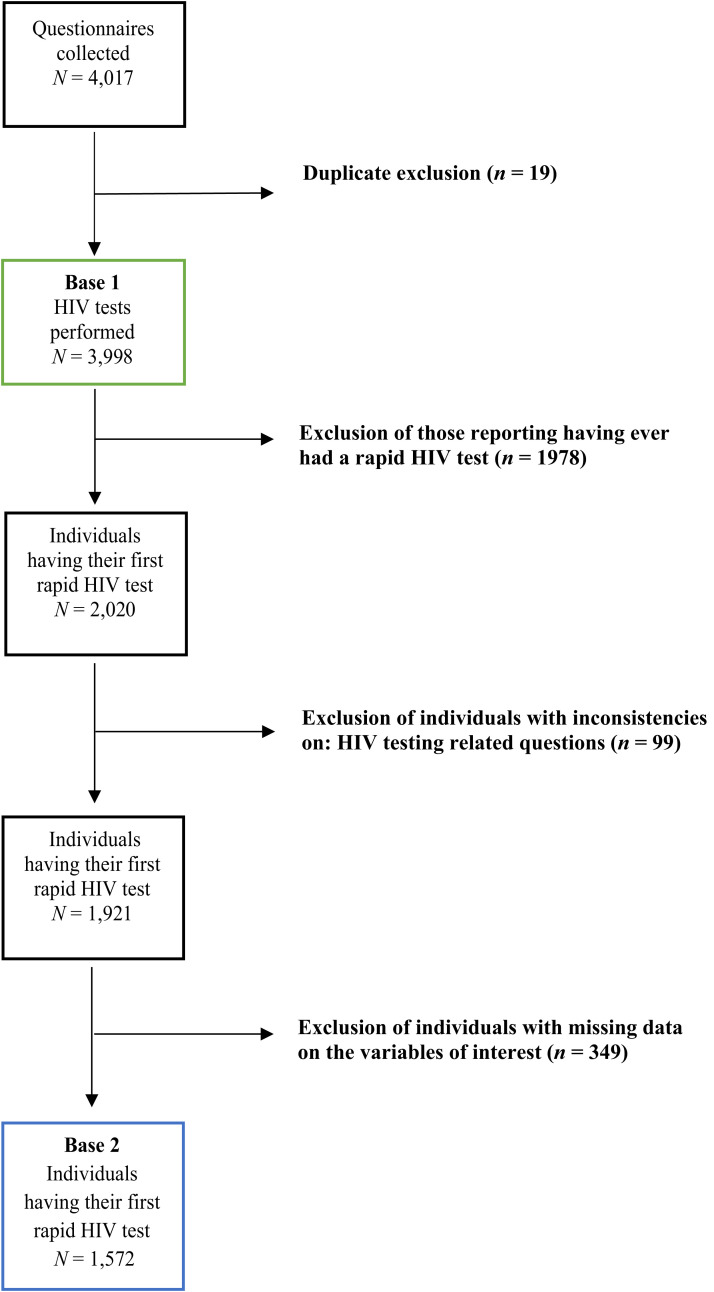
Flowchart of database selection for 1) evolution of the HIV test (base 1) and 2) first HIV rapid test respondents (base 2).

Over the 3-year program, 445 interventions were carried out in 91 different places between July 31, 2015 and April 11, 2018, resulting in 4,017 questionnaires and 3,998 rapid HIV tests performed (Figure 1). Of the 3,998 tests, 710 were first-time HIV testers (17.8%), and 245 were HIV positive (6.1%): 204 MSM, 12 FSWs, and 29 nonkey population. Among the 245 who tested HIV positive, 207 (84.5%) were tested for the first time.

**Figure 2.**
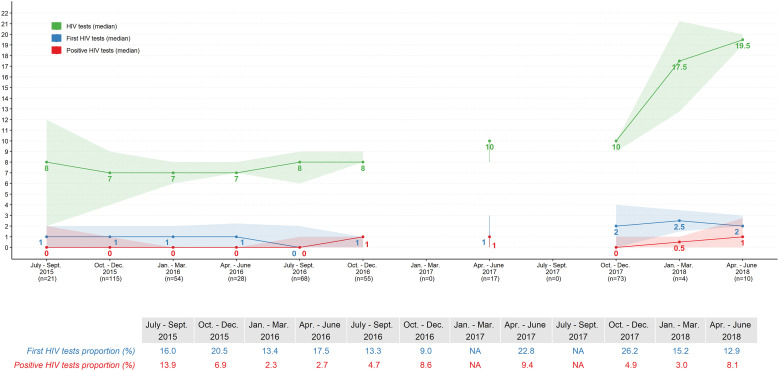
Evolution of the numbers of all tests, first HIV tests, and positive HIV tests performed by individuals in Haiti from 2015 to 2018 (*n* = 445). Note that interquartile ranges have been computed. *n* = number of actions; NA = not applicable.

Overall, a median of eight individuals (minimum to maximum: 1–34) was tested per intervention (Figure 2), including a median of one first-time HIV-tested individual (minimum to maximum: 0–20) and zero individuals (minimum to maximum: 0–9) who tested HIV positive. At least one individual tested positive for HIV in 31.9% (*n* = 142/445) of the total interventions. During the 3-year program, the evolution of the number of individuals tested per intervention increased over the study period, although this was not significant given the 0.05 threshold (linear regression coefficient: 0.75, 95% CI [−0.14 to 1.64], *P* = 0.09). The evolution of the number of HIV-positive individuals tested also increased over the time (0.07, 95% CI [−0.01 to 0.15], *P* = 0.07). In addition, the evolution of the number of first-time testers increased significantly over the study period (0.13, 95% CI [0.03–0.24], *P* = 0.02). First-time testers were more likely to be found through interventions conducted in the community NGO settings, streets, or cartels compared with other places (median first-time tester numbers per intervention: one versus zero, *P* <0.001). Furthermore, during the program period, there were no tests conducted during January to March 2017 and July to September 2017 (Figure 2).

When considering tests conducted between MSM and FSWs, the evolution of the median numbers of tested individuals per intervention increased among MSM (0.71, 95% CI [−0.20 to 1.62], *P* = 0.11) and FSWs (1.06, 95% CI [0.16–1.97], *P* = 0.03) (Supplemental Figure 1). Compared with FSWs, MSM were more likely to be a first-time tester (19.8% versus 11.1%, respectively, *P* <0.001) and to test HIV positive (7.4% versus 1.9%, respectively, *P* <0.001).

Regarding changes in the proportions of first-time HIV tests (Figure 2), the percentages of individuals accepting their first HIV test were always above 10% by trimester, except for in the period from October to December 2016 (9%). This proportion exceeded 20% for three trimesters (October to December 2015, April to June 2017, and October to December 2017). The proportion of HIV-positive tests was 14% for the July to September 2015 period and ranged from 2% to 9% depending on the period. When considering MSM and FSW proportions alone, MSM were more likely to be tested for the first time (20% versus 11% overall) or to test positive for HIV (7% versus 2% overall) (Supplemental Table 1).

### Characteristics of the first community-based HIV testing users.

Among the 3,998 tested individuals, 2,020 individuals were tested through RDT for the first time (Figure 1). Of these, we excluded 99 individuals with inconsistencies related to their testing history and 349 individuals with missing data on one of the variables of interest. Comparison of individual samples before and after exclusion of individuals with missing data is available in Supplemental Table 2. The selection of individuals reveals a higher proportion of MSM (77% versus 75%) and a lower proportion of the nontarget population (8% versus 11%). Individuals with missing data on condom use at last intercourse variable (not applicable = 93) and reported STI in the last 12 months variable (not applicable = 439) were kept in the analysis. Thus, a total of 1,572 first-time RDT testers were included in our analysis.

In this sample, 1,216 MSM were tested from July 31, 2015 to April 11, 2018 (77.4%), 235 FSWs were tested from September 18, 2015 to February 11, 2018 (15.0%), and 121 nonkey population members were tested from September 11, 2015 to February 9, 2018 (7.6%), including 71 men (58.7%) and 50 women (41.3%) (Table 1).

**Table 1:** Sociodemographic characteristics, practices, and HIV testing for men who have sex with men, female sex workers, and the nonkey population who performed a first rapid HIV test for the first time in Haiti from 2015 to 2018 (*N* = 1,572)

Characteristics Categories	MSM (*N* = 1,216), *n* (%)	FSW (*N* = 235), *n* (%)	Nonkey Population (*N* = 121), *n* (%)	Overall (*N* = 1,572), *n* (%)
Sociodemographic				
Sex				
Men	1,216 (100.0)	0 (0.0)	71 (58.7)	1,287 (81.9)
Women	0 (0.0)	235 (100.0)	50 (41.3)	285 (18.1)
Age (years)				
≤20	234 (19.2)	33 (14.0)	23 (19.0)	290 (18.4)
21–29	678 (55.8)	141 (60.0)	69 (57.0)	888 (56.5)
≥30	304 (25.0)	61 (26.0)	29 (24.0)	394 (25.1)
Region				
Capital	782 (64.3)	151 (64.3)	76 (62.8)	1,009 (64.2)
Region	434 (35.7)	84 (35.7)	45 (37.2)	563 (35.8)
Reported financial resources				
None or not enough	694 (57.1)	131 (55.7)	57 (47.1)	882 (56.1)
Sufficient but need to be careful	142 (11.7)	32 (13.6)	18 (14.9)	192 (12.2)
Sufficient or financially comfortable	380 (31.2)	72 (30.6)	46 (38.0)	498 (31.7)
Have sexual relations with				
Men or women	452 (37.2)	56 (23.8)	82 (67.8)	590 (37.5)
Men only	764 (62.8)	179 (76.2)	39 (32.2)	982 (62.5)
HIV risk exposure and perceptions				
No. of partners (≤6 months)				
Fewer than 10 partners	879 (72.3)	25 (10.6)	90 (74.4)	994 (63.2)
More than 10 partners	337 (27.7)	210 (89.4)	31 (25.6)	578 (36.8)
Transactional sex (≤12 months)				
No	912 (75.0)	0 (0.0)	109 (90.1)	1,021 (64.9)
Yes	304 (25.0)	235 (100.0)	12 (9.9)	551 (35.1)
Psychoactive drug use (≤12 months)				
No	1,039 (85.4)	169 (71.9)	95 (78.5)	1,303 (82.9)
Yes	177 (14.6)	66 (28.1)	26 (21.5)	269 (17.1)
Condom use at last intercourse				
Yes	850 (69.9)	138 (58.7)	68 (56.2)	1,056 (67.2)
No or missing	366 (30.1)	97 (41.3)	53 (43.8)	516 (32.8)
Perception of HIV risk				
No or low	915 (75.2)	70 (29.8)	71 (58.7)	1,056 (67.2)
High or very high	301 (24.8)	165 (70.2)	50 (41.3)	516 (32.8)
STI (≤12 months)				
No	627 (51.6)	153 (65.1)	60 (49.6)	840 (53.4)
Yes	136 (11.2)	44 (18.7)	15 (12.4)	195 (12.4)
Missing or do not know	453 (37.3)	38 (16.2)	46 (38.0)	537 (34.2)
Testing				
Ever been tested for HIV				
Yes	823 (67.7)	183 (77.9)	77 (63.6)	1,083 (68.9)
No	393 (32.3)	52 (22.1)	44 (36.4)	489 (31.1)
Type of location				
Cartel	761 (62.6)	105 (44.7)	58 (47.9)	924 (58.8)
Association room	213 (17.5)	28 (11.9)	27 (22.3)	268 (17.0)
Street	104 (8.6)	36 (15.3)	18 (14.9)	158 (10.1)
Other	138 (11.3)	66 (28.1)	18 (14.9)	222 (14.1)

FSW = female sex worker; MSM = men who have sex with men; STI = sexually transmitted infection.

In the overall sample, the median age was 25 years old (interquartile range: 21–29), more than half of the individuals (56.1%) reported having very little or no financial means, and 64.2% resided in the Western Department where the capital Port-au-Prince is located.

All three groups reported sexual HIV exposure, such as transactional sex (25.0% of MSM, 100% of FSWs, and 9.9% of the nonkey population), a high number of sexual partners (i.e., >10 sexual partners in the last 6 months; 27.7% of MSM, 88.4% of FSWs, and 25.6% of the nonkey population), lack of condom use at last intercourse (30.1% of MSM, 41.3% of FSWs, and 43.8% of the nonkey population), and reported STI (11.2% of MSM, 18.7% of FSWs, and 12.4% of the nonkey population). Drug use was reported by one sixth to one quarter of individuals depending on the group in our sample (14.6% of MSM, 28.1% of FSWs, and 21.5% of the nonkey population). MSM were more likely to test positive for HIV (6.2% of MSM, 1.3% of FSWs, and 2.5% of the nonkey population, *P* = 0.003).

### Factors associated with never been HIV tested before among program users.

In our sample, 489 had their first HIV test (31.1%) within 393 MSM (32.3%), 52 FSWs (22.1%), and 44 nonkey population members (36.4%) (Table 2). Factors associated with the first HIV test after adjustment in the community-based program for each group are shown in Table 3.

**Table 2:** Univariate analysis of people who have never been tested for HIV among men who have sex with men, female sex workers, and the nonkey population with multiple criteria (sociodemographic characteristics, practices, and HIV testing) in Haiti from 2015 to 2018 (*N* = 1,572)

Characteristics Categories	MSM (393/1,216), *n/N* (%)	*P*-Value*	FSW (52/235), *n/N* (%)	*P*-Value*	Nonkey Population (44/121), *n/N* (%)	*P*-Value*
Sociodemographic				
Sex						0.317
Men					29/71 (40.8)	
Women					15/50 (30.0)	
Age (years)		**<0.001**		**0.198**		**<0.001**
≤20	124/234 (53.0)		8/33 (24.2)		17/23 (73.9)	
21–29	218/678 (32.2)		26/141 (18.4)		25/69 (36.2)	
≥30	51/304 (16.8)		18/61 (29.5)		2/29 (6.9)	
Region		**0.072**		0.235		0.349
Capital	279/782 (35.7)		38/151 (25.2)		30/76 (39.5)	
Region	114/434 (26.3)		14/84 (16.7)		14/45 (31.1)	
Reported financial resources		0.217		0.388		0.265
None or not enough	242/694 (34.9)		24/131 (18.3)		24/57 (42.1)	
Sufficient but need to be careful	45/142 (31.7)		9/32 (28.1)		8/18 (44.4)	
Sufficient or financially comfortable	106/380 (27.9)		19/72 (26.4)		12/46 (26.1)	
Have sexual relations with		>0.995		0.833		0.265
Men or women	146/452 (32.3)		13/56 (23.2)		33/82 (40.2)	
Men only	247/764 (32.3)		39/179 (21.8)		11/39 (28.2)	
HIV risk exposure and perceptions				
No. of partners (≤6 months)		**0.149**		0.865		0.399
Fewer than 10 partners	297/879 (33.8)		5/25 (20.0)		35/90 (38.9)	
More than 10 partners	96/337 (28.5)		47/210 (22.4)		9/31 (29.0)	
Transactional sex (≤12 months)		**0.003**				0.815
No	322/912 (35.3)				40/109 (36.7)	
Yes	71/304 (23.4)				4/12 (33.3)	
Psychoactive drug use (≤12 months)		0.869		**0.050**		**0.186**
No	337/1,039 (32.4)		44/169 (26.0)		37/95 (38.9)	
Yes	56/177 (31.6)		8/66 (12.1)		7/26 (26.9)	
Condom use at last intercourse		**<0.001**		**0.026**		**0.025**
Yes	237/850 (27.9)		21/138 (15.2)		18/68 (26.5)	
No or missing	156/366 (42.6)		31/97 (32.0)		26/53 (49.1)	
Perception of HIV risk		**0.002**		0.690		0.460
No or low	269/915 (29.4)		17/70 (24.3)		24/71 (33.8)	
High or very high	124/301 (41.2)		35/165 (21.2)		20/50 (40.0)	
STI (≤12 months)		**0.009**		0.534		0.782
No	240/627 (38.3)		31/153 (20.3)		23/60 (38.3)	
Yes	45/136 (33.1)		12/44 (27.3)		6/15 (40.0)	
Missing or do not know	108/453 (23.8)		9/38 (23.7)		15/46 (32.6)	
Testing				
Type of location		0.765		0.599		0.958
Cartel	251/761 (33.0)		27/105 (25.7)		22/58 (37.9)	
Association room	58/213 (27.2)		4/28 (14.3)		9/27 (33.3)	
Street	36/104 (34.6)		8/36 (22.2)		6/18 (33.3)	
Other	48/138 (34.8)		13/66 (19.7)		7/18 (38.9)	

FSW = female sex worker; MSM = men who have sex with men; STI = sexually transmitted infection.

* The χ^2^ test with the Rao and Scott second-order correction was used.

Significant *P*-values (>0.200) are shown in bold.

**Table 3:** Multivariate analysis of people who have never been tested for HIV among men who have sex with men, female sex workers, and the nonkey population with multiple criteria (sociodemographic characteristics, practices, etc.) in Haiti from 2015 to 2018 (*N* = 1,572)

	MSM Multivariable Model					FSW Multivariable Model					Nonkey Population Multivariable Model				
Characteristics	% MSM	aOR	95% CI	*P*-Value	aGVIF	% FSW	aOR	95% CI	*P*-Value	aGVIF	% Others	aOR	95% CI	*P*-Value	aGVIF
Age (years)				**<0.001**	1.0				**0.046**	1.2				**<0.001**	1.1
21–29	32.2	1.00	–			18.4	1.00	–			36.2	1.00	–		
≤20	53.0	2.79	1.82–4.28	**<0.001**		24.2	1.93	0.73–5.08	0.197		73.9	4.82	2.03–11.5	**0.001**	
≥30	16.8	0.45	0.34–0.59	**<0.001**		29.5	1.94	1.08–3.47	**0.037**		6.9	0.14	0.03–0.60	**0.013**	
Region				0.309	1.2										
Capital	35.7	1.00	–												
Region	26.3	0.78	0.48–1.26	0.309											
Transactional sex (≤12 months)	23.4			**<0.001**	1.2										
No		1.00	–												
Yes		0.47	0.33–0.68	**<0.001**											
Condom use at last intercourse				**<0.001**	1.2				**0.009**	1.3				0.063	1.2
Yes	27.9	1.00	–			15.2	1.00	–			26.5	1.00	–		
No or missing	42.6	1.99	1.43–2.77	**<0.001**		32.0	2.82	1.30–6.11	**0.015**		49.1	2.30	0.96–5.53	0.073	
Perception of HIV risk				**<0.001**	1.0										
No or low	29.4	1.00	–												
High or very high	41.2	1.81	1.32–2.46	**<0.001**											
STI (≤12 months)				**0.008**	1.2										
No	38.3	1.00	–												
Yes	33.1	0.72	0.36–1.46	0.378											
Missing or do not know	23.8	0.52	0.34–0.79	**0.003**											
Psychoactive drug use (≤12 months)						12.1			**0.045**	1.4	26.9				
No							1.00	–							
Yes							0.41	0.17–0.98	0.057						

aGVIF = adjusted generalized variance inflation factor; aOR = adjusted odds ratio; FSW = female sex worker; MSM = men who have sex with men; STI = sexually transmitted infection.

Significant *P*-values (>0.050) are shown in bold.

Never previously tested MSM were less likely to test positive for HIV (16.0% versus 33.4%, *P* <0.001) but not FSWs and nonkey population individuals (33.3% versus 22.0%, *P* = 0.65 and 66.7% versus 35.6%, *P* = 0.29, respectively). Among MSM, younger age (younger than 20 years old; 3.0%, *P* <0.001), lack of condom use at last intercourse (42.6%, *P* <0.001), and perception of being at high risk for HIV (41.2%, *P* = 0.002) were independently associated with the first HIV test. In the last 12 months, transactional sex (23.4%, *P* = 0.003) was also independently associated with the first HIV test in a negative way. These associations were still significantly associated in the multivariable models (Table 3).

Among FSWs, older age (older than 30 years old; 29.5%, *P* = 0.20) and lack of condom use at last intercourse (32.0%, *P* = 0.026) were independently associated with being HIV tested for the first time. Recent (<12 months) psychoactive drugs use (12.1%, *P* = 0.050) was significantly less associated with being HIV tested for the first time. These associations were still significantly associated in the multivariable models (Table 3).

Among the nonkey population, younger age (younger than 20 years old; 30.0%, *P* <0.001) was independently associated with the first HIV test. This association was still significant in the multivariable models (Table 3).

Regardless of the group, there were no significant differences found in the number of reported sexual partners between ever and never previously tested individuals.

## DISCUSSION

Our results show that the community-based HIV testing program considered in our study was able to reach both HIV-positive (6.1%) and previously untested (17.8%) populations in Haiti, with relative stability over the 3 years of the program in terms of the number of tests performed and the number of HIV-positive individuals identified. Of those who tested positive, about a quarter (25.5%) had never been tested for HIV. In particular, the HIV prevalence of testing among MSM was 7.4%, which was under the MSM HIV national prevalence of 12.9%.^1^ This difference may be explained by the fact that MSM tested in traditional settings, such as laboratories, are more likely to seek testing only when experiencing symptoms or after high-risk behaviors, whereas our community-based program also reaches MSM who are not necessarily at risk. These results underline the relevance of maintaining such a program in the Haiti context.

Our study has shown the capacity of the community-based HIV testing program for reaching HIV-positive individuals over the 3-year program as well as confirming results found in studies conducted elsewhere. For example, in Africa and Asia, research has shown that a community-based testing program delivered over 3 years is able to engage and reach HIV-positive individuals as well as increasing repeated testing amongst them.^21^ Another study reported a greater HIV-positive rate than the standard of care.^22^ In the European context, a 6-month community-based testing program in Italy highlighted an HIV-positive rate around 1%, with a rate of 45% of previously untested individuals.^23^ In Barcelona, an MSM community-based center offering HIV testing was found to be cost-effective over time and represented 36% of all new HIV diagnoses among MSM in the Catalonia region during 2009–2011.^24^ Thus, our results confirm the efficacy of community-based approaches for key populations and strongly advocate for the development of community-based programs in general.

Our study reveals an inconsistent number of interventions conducted over the program period (the January to March 2017 and July to September 2017 periods). This aspect was reported by the program leader as linked to the delays in the disbursement of funds, which disrupted intervention conduction. Nonregularity and a complex disbursement process have been found to have a major impact with regard to the frequency of the interventions as well as the overall quality.^25^^,^^26^ Additionally, the program leader reported blockages from the National Public Health Laboratory on the evaluation of the effectiveness and medical procedures of community-based screening midway during the project. Although the no-significance of the trend may be because of a lack of statistical power and even if the trend proves to be nonsignificant, these screening activities remain justified by the fact that the program’s effectiveness remains stable and does not decrease over time.

Our results have shown that the community-based HIV testing program was able to reach highly HIV-exposed MSM and FSWs as well as nonkey population members for whom a testing offer is clearly recommended. The capacity of community-based HIV testing programs to reach and engage with HIV-exposed populations has been found in other contexts.^9^^,^^15^ In addition, in our study, almost half of the participants in the program had never been tested before, which suggests that the program reached a population that does not access HIV testing through other venues. Community-based HIV testing strategies are particularly effective at engaging with HIV-exposed individuals who do not want to or who cannot access the usual health care facilities.^27^^,^^28^

Our results showed that male and younger populations were more likely to have never been tested for HIV before their participation in the program. Male and young populations are well known as having lower access to HIV testing in particular in sub-Saharan Africa, and in the Caribbean,^1^ community-based approaches have been shown to be particularly effective when targeting these two populations.^21^^,^^22^

More than half of the program participants reported no or very limited financial resources. Although HIV testing is free in Haiti,^29^ transport costs to access the nearest HIV center can be a barrier, especially in remote or rural areas.^2^^,^^4^^,^^30^

Although those who had been tested for the first time are less exposed in terms of transactional sex and psychoactive drug consumption than those who have already been tested, they are still considerably exposed in view of the use of condoms at last intercourse. Association between the absence of condom use at last intercourse and first HIV test is widely documented within the extant literature.^2^^,^^4^^,^^6^^,^^31^^,^^32^ It could suggest that nontested individuals had worse access to HIV prevention, or on the contrary, it could underline that people who are tested usually benefit from HIV prevention services that prompt them to adopt protective behavior.

Our findings indicate that a significantly higher proportion of first-time HIV testers were tested within the capital region of Port-au-Prince compared with other departments in Haiti, with a notable difference observed among MSM. This trend could suggest a greater need for expanding testing services in this region. In contrast, no significant differences were observed regarding the intervention sites of the testing program. Finally, first-time testers appeared to perceive themselves as being at risk or at high risk of HIV infection, particularly among MSM, highlighting the need for enhanced education on prevention and improved access to primary HIV prevention services.

Our study has limitations that we wish to acknowledge. First, only program participants who reported having never been previously tested by RDT were considered to avoid cases with more than one questionnaire per individual for those who had tested several times throughout the program. Thus, individuals who undergone RDT through other NGOs or clinical facilities may have been excluded, even though we are not aware of any other NGOs offering this service in Haiti during the study period. Classifying missing data as “no” for condom use at last intercourse and for reported STI may have underestimated condom use and whether people had contracted one or more STIs in the last 12 months. Excluding individuals because of missing data revealed only one significant difference: a slight increase in the number of MSM and a slight decrease in the number of individuals from the nonkey population. This could have an influence on an overrepresentation of MSM and an underrepresentation of the nonkey population, but as we studied our three population groups in a differentiated way, this should not induce a selection bias in our study. Second, responses to the questionnaires were self-reported, and some sexual behaviors, such as the number of sexual partners or lack of condom use during the last intercourse, may have been underestimated as a result of desirable bias. Third, our program did not include data collection on linkage to care after HIV positivity in the community-based program. Every participant testing positive for HIV was linked to the nearest clinic or facilities, but no follow-up was collected for confidentiality reasons. Linkage to care has, however, been demonstrated as effective in community-based testing programs elsewhere^33^^,^^34^ and specifically, in community-based centers.^24^

We believe that our intervention could be successfully implemented in other similar contexts. The involvement of trained community peer educators was a key factor in the success of this intervention. These educators were able to create a friendly environment that significantly improved the uptake of community-based HIV testing, a trend also observed in other contexts.^22^^,^^35^^,^^36^ This approach proved effective despite the legal and societal stigmatization of MSM in Haiti.^37^^,^^38^ In highly stigmatized contexts, community-based interventions are often the only viable means of reaching marginalized populations. Additionally, the program was implemented in places chosen by the community associations and those well known to be visited by MSM and FSWs across the country to be sure to cover a large part of this population.^39^ This implementation by people from the community could have contributed to better targeting and acceptance of the program.

To maximize the accuracy of our results, we took into account the cluster effect that may be associated with program testing locations in our analyses.^20^

In Haïti, the community-based HIV testing approach could be strengthened with the introduction of the distribution of HIV self-testing, which has been shown to be effective with reaching other at-risk populations, such as clients of FSWs or MSM who do not visit community-based venues.^40^

## CONCLUSION

This study of a community-based HIV testing program shows relative stability with engaging HIV-exposed populations, with consistent reach of those who test for the first time and those who discover their HIV status through the program. The community component of the program thus allows for better targeting and acceptance by key populations. This confirms the importance of maintaining, developing, and sustaining the resources allocated to community-based screening programs as part of the reduction of the undiagnosed epidemic in addition to routine HIV testing services in Haiti.

## Supplemental Materials

10.4269/ajtmh.24-0391 Supplemental Materials
