# Supplementary material for: Temporal Trends and Characteristics of Community-Based HIV Testing: First Community-Based Testing Program Results of Men Who Had Sex with Men and Female Sex Workers in Haiti, 2015–2018
Source: Am J Trop Med Hyg. 2025 Jul 8;113(3):564–72. doi: 10.4269/ajtmh.24-0391 (PMC12410271; doi:10.4269/ajtmh.24-0391)
Supplement: Supplemental Materials [file tpmd240391.SD1.pdf]

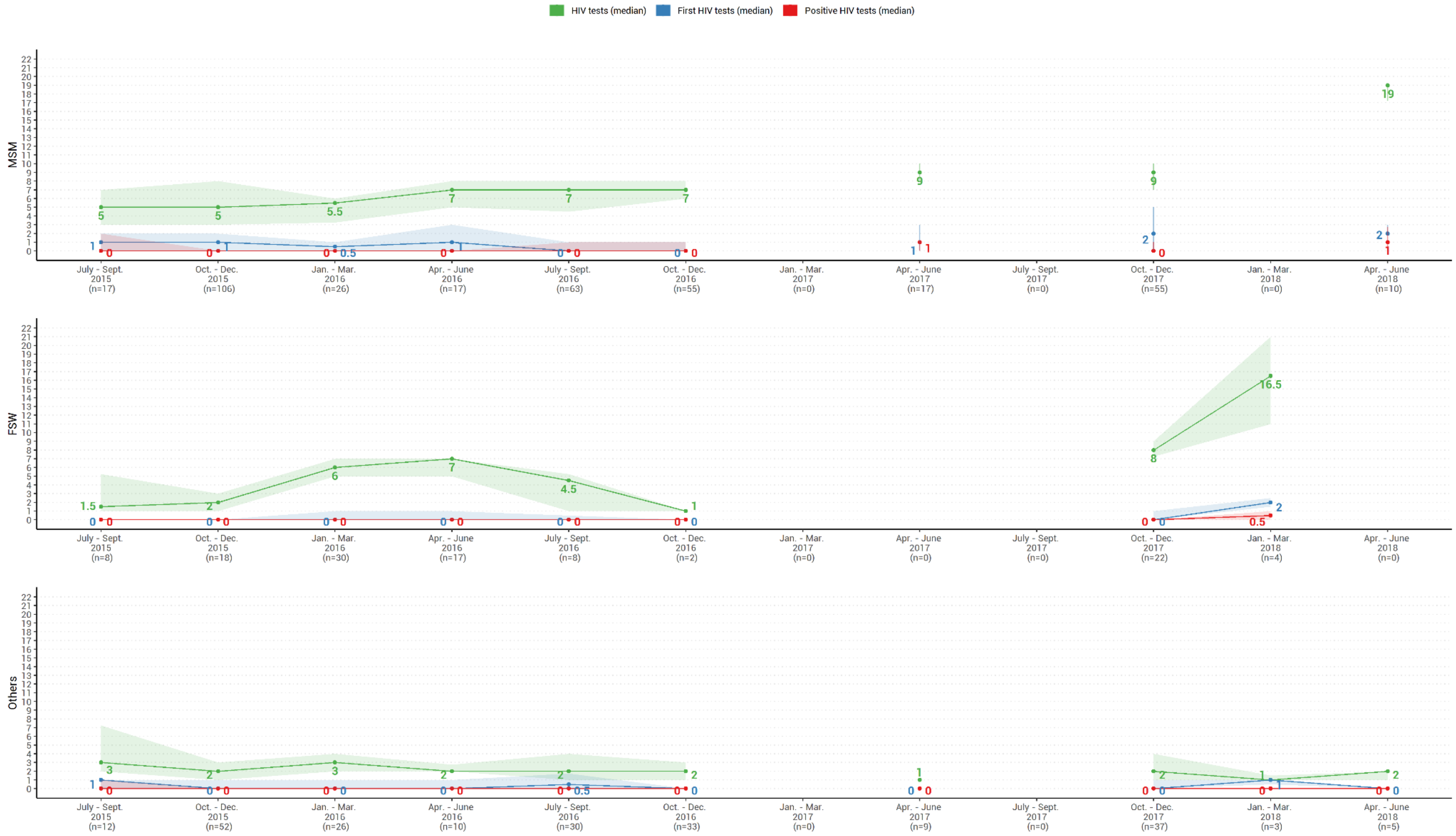

Supplementary data - Figure 1: Evolution of number of all tests, first HIV tests and positive HIV tests performed by population, Haiti, 2015-2018 (n=445), IQR has been computed

MSM: Men who have sex with men; FSW: Female Sex Workers; Others: other non-MSM and non-FSW population

Supplementary Data Table 1. Evolution of proportion of first HIV tests and positive HIV tests performed by population, Haiti, 2015-2018 (n=445)

	July - Sept. 2015	Oct. - Dec. 2015	Jan. - Mar. 2016	Apr. - June 2016	July - Sept. 2016	Oct. - Dec. 2016	Jan. - Mar. 2017	Apr. - June 2017	July - Sept. 2017	Oct. - Dec. 2017	Jan. - Mar. 2018	Apr. - June 2018
**MSM**												
Number of intervention (n)	17	106	26	17	63	55	0	17	0	55	0	10
First HIV tests proportion (%)	*19.8*	*25.1*	*12.6*	*28.0*	*10.5*	*9.0*	*NA*	*22.9*	*NA*	*31.0*	*NA*	*14.0*
Positive HIV tests proportion (%)	17.8	7.7	4.9	4.9	5.2	8.6	NA	9.5	NA	5.9	NA	8.5
**FSW**												
Number of intervention (n)	8	18	30	17	8	2	0	0	0	22	4	0
First HIV tests proportion (%)	3.2	12.0	12.3	9.7	13.3	0.0	NA	NA	NA	11.0	13.6	NA
Positive HIV tests proportion (%)	0.0	7.5	0.0	0.8	3.3	0.0	NA	NA	NA	2.3	3.2	NA
**Others**												
Number of intervention (n)	12	52	26	10	30	33	0	9	0	37	3	5
First HIV tests proportion (%)	16.4	26.6	17.5	17.9	25.5	10.0	NA	25.0	NA	29.5	50.0	0.0
Positive HIV tests proportion (%)	14.5	2.5	2.5	3.3	3.0	8.4	NA	8.3	NA	3.7	0.0	0.0

Supplementary Data Table 2: Comparison of sociodemographic characteristics, practices and HIV testing of individuals before and after missing data exclusion, Haiti, 2015-2018 (n=1572)

Characteristics Categories	Characteristics	Before individuals exclusion, n (%) N=1921	After individuals exclusion, n (%) N=1572	p-value[1]
	**Target population**			**0.006**
	MSM	1,396 (74.5)	1,216 (77.4)	
	FSW	274 (14.6)	235 (14.9)	
	Non-key population	204 (10.9)	121 (7.7)	
	Missing	47	0	
	**Gender**			0.602
	Men	1,518 (81.2)	1,287 (81.9)	
	Women	352 (18.8)	285 (18.1)	
**Sociodemographic**	Missing	51	0	
	**Age**			0.828
	<= 20 years old	363 (19.0)	290 (18.4)	
	21 - 29 years old	1,080 (56.7)	888 (56.5)	
	>= 30 years old	463 (24.3)	394 (25.1)	
	Missing	15	0	
	**Region**			0.629
	Capital	1,214 (63.4)	1,009 (64.2)	
	Region	701 (36.6)	563 (35.8)	
	Missing	6	0	
	**Reported financial resources**			0.694
	None or not enough	1,057 (55.9)	882 (56.1)	
	Sufficient, but need to be careful	216 (11.4)	192 (12.2)	
	Sufficient or financially comfortable	618 (32.7)	498 (31.7)	
	Missing	30	0	
	**Have sexual relations**			0.758
	Men or women	697 (38.0)	590 (37.5)	
**HIV risk exposure and perceptions**	Men only	1,135 (62.0)	982 (62.5)	
	Missing	89	0	
	**Number of partners (<=6 months)**			0.860
	Less than 10 partners	1,139 (63.5)	994 (63.2)	
	More than 10 partners	654 (36.5)	578 (36.8)	
	Missing	128	0	
	**Transactional sex (<=2 months)**			0.888
	No	1,241 (65.2)	1,021 (64.9)	
	Yes	663 (34.8)	551 (35.1)	
	Missing	17	0	
	**Psychoactive drug use (<=12 months)**			0.593

	No	1,551 (82.2)	1,303 (82.9)	
	Yes	336 (17.8)	269 (17.1)	
	Missing	34	0	
	**Condom use at last intercourse**			0.487
	Yes	1,269 (66.1)	1,056 (67.2)	
	No or missing	652 (33.9)	516 (32.8)	
	**Perception of HIV risk**			0.499
	No or low	1,253 (66.1)	1,056 (67.2)	
	High or very high	643 (33.9)	516 (32.8)	
	Missing	25	0	
	**STI (<=12 months)**			0.927
**Testing**	No	1,032 (53.7)	840 (53.4)	
	Yes	230 (12.0)	195 (12.4)	
	Missing or don't know	659 (34.3)	537 (34.2)	
	**Ever been tested for HIV**			0.658
	Yes	1,310 (68.2)	1,083 (68.9)	
	No	611 (31.8)	489 (31.1)	
	**Type of location**			0.700
	Cartel	1,087 (56.9)	924 (58.8)	
	Association room	336 (17.6)	268 (17.0)	
	Street	207 (10.8)	158 (10.1)	
	Non-key population	282 (14.7)	222 (14.1)	
	Missing	9	0	

[1]*Pearson's Chi-squared test*
